# Persistent Left Superior Vena Cava Mimicking Central Venous Catheter Malposition After Left Internal Jugular Access: A Case Report

**DOI:** 10.1155/cria/7087135

**Published:** 2026-07-19

**Authors:** Daniel A. Navid, Nasir Notta, Jason M. Buehler

**Affiliations:** ^1^ Department of Anesthesiology, University of Tennessee Medical Center, Knoxville, Tennessee, USA, tennessee.edu; ^2^ Department of Internal Medicine, University of Tennessee Medical Center, Knoxville, Tennessee, USA, tennessee.edu

## Abstract

Persistent left superior vena cava (PLSVC) is the most common congenital thoracic venous anomaly and is usually clinically silent. We report a critically ill patient in whom PLSVC was identified after left internal jugular central venous catheter placement. Chest radiography showed a vertically oriented catheter along the left mediastinum without expected midline crossover, raising concern for malposition. Review of prior imaging confirmed PLSVC draining into the coronary sinus. Recognition of this variant prevented unnecessary catheter removal and repeat instrumentation, underscoring the importance of correlating procedural findings with imaging during left‐sided venous access.

## 1. Introduction

Persistent left superior vena cava (PLSVC) is the most common congenital anomaly of the thoracic venous system, with a reported prevalence of less than 0.5% in the general population and up to 4% in patients with congenital heart disease [1]. PLSVC results from failure of regression of the embryologic left anterior cardinal vein, resulting in persistence of a left‐sided superior vena cava [1, 2]. In the absence of associated congenital cardiac abnormalities, a PLSVC most commonly drains into the coronary sinus and is typically hemodynamically insignificant. However, venous drainage may be variable and, less commonly, may drain directly into the left atrium [1, 2].

Because PLSVC is usually asymptomatic, it is often discovered incidentally during cardiovascular imaging or invasive procedures performed for unrelated indications. Despite its benign nature, PLSVC has important implications during central venous catheterization, particularly when left‐sided venous access is utilized. When a catheter is placed via the left internal jugular or subclavian vein, a PLSVC may result in an atypical left‐sided mediastinal catheter course on postprocedure imaging, potentially mimicking catheter malposition or unintended arterial cannulation [3]. Failure to recognize this anatomic variant may lead to unnecessary catheter removal, repeat instrumentation, or additional imaging.

## 2. Case Description

A 62‐year‐old male presented with hypothermia and septic shock, accompanied by altered mental status. Due to progressive encephalopathy and inability to protect his airway, the patient was intubated and mechanically ventilated. Despite initial resuscitation, he developed persistent hypotension requiring escalating doses of norepinephrine.

Given the need for ongoing vasopressor support, a central venous catheter was placed in the left internal jugular vein. The procedure was performed under ultrasound guidance using standard sterile technique. Venous access was confirmed by nonpulsatile dark blood return, and the catheter was advanced without resistance. All ports aspirated and flushed easily, and there were no immediate procedural complications.

Postprocedure chest radiography demonstrated an unexpected left‐sided mediastinal course of the catheter, tracking along the lateral border of the left mediastinum rather than crossing midline (Figure 1). This atypical trajectory raised initial concern for possible catheter malposition, prompting radiology consultation for further evaluation. Review of the available chest CT was performed to correlate the catheter course with cross‐sectional venous anatomy. On coronal imaging, a duplicated superior vena cava was identified, with a left‐sided venous channel descending along the left mediastinal border in the same expected location as the catheter course seen on chest radiography (Figure 2). On axial images, this vessel was separate from the aorta and pulmonary artery and coursed inferiorly toward the coronary sinus and right atrium. Based on these CT landmarks, the catheter tip projected within the distal PLSVC drainage pathway near the coronary sinus rather than within an artery, pleural space, or unintended small venous branch. No additional catheter‐directed venography, contrast injection through the catheter, or transesophageal echocardiography was obtained because CT anatomy and procedural findings confirmed an acceptable venous course without evidence of catheter‐related complication. After radiology review, the catheter was retained for ongoing central venous access and vasopressor administration. The catheter remained in place for the remainder of the patient’s ICU stay and was not associated with catheter‐related arrhythmias, infusion‐related complications, or hemodynamic instability attributable to catheter position. A summary of the patient’s course is provided in Table 1.

**FIGURE 1:**
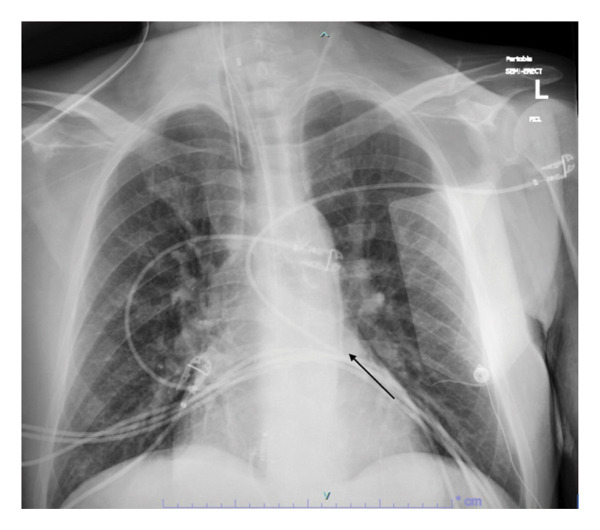
Portable anteroposterior chest radiograph obtained following left internal jugular central venous catheter placement demonstrates an atypical left‐sided mediastinal course of the catheter (arrow). The catheter descends along the left mediastinal border without crossing the midline.

**FIGURE 2:**
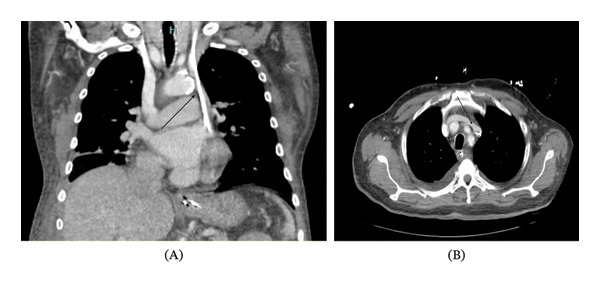
Computed tomography demonstrating a persistent left superior vena cava. (A) Coronal image showing a vertically oriented venous structure coursing along the left mediastinal border (arrow). (B) Axial image demonstrating the left‐sided superior vena cava (arrow), correlating with the atypical catheter trajectory observed on chest radiography.

**TABLE 1:** Clinical timeline.

Time point	Event
Presentation	Patient presented with hypothermia, septic shock, and altered mental status.
Early ICU course	Patient was intubated for progressive encephalopathy and required norepinephrine for persistent hypotension.
Central venous access	Left internal jugular central venous catheter was placed; chest radiography demonstrated an atypical left‐sided mediastinal catheter course.
Diagnostic clarification and outcome	Admission CT demonstrated a persistent left superior vena cava draining toward the coronary sinus. The catheter was retained and used without catheter‐related arrhythmia, infusion complication, or hemodynamic instability.

## 3. Discussion

PLSVC is a congenital venous anomaly that results from persistence of the left anterior cardinal vein. The vessel receives venous return from the left head, neck, and upper extremity and descends along the left mediastinum rather than following the usual pathway across the midline through the left brachiocephalic vein into the right‐sided superior vena cava. In most patients, PLSVC drains into the coronary sinus and then the right atrium, which explains why the anomaly is often clinically silent and hemodynamically insignificant. Coronary sinus dilation may be seen when PLSVC drains through this pathway and can serve as an imaging clue. Less commonly, PLSVC drains directly into the left atrium or through an unroofed coronary sinus, creating the potential for right‐to‐left shunting [4]. Bedside transthoracic echocardiography was performed during the shock state; however, coronary sinus dilation was not documented in the available echocardiographic interpretation in this case. During central venous catheterization via the left internal jugular or subclavian vein, PLSVC may produce an atypical catheter trajectory along the left mediastinum on postprocedure chest radiography. Under normal anatomy, catheters placed from the left internal jugular vein cross the midline via the left brachiocephalic vein before descending into the right‐sided superior vena cava. In the presence of PLSVC, this expected midline crossover is absent, resulting in a vertically oriented left‐sided catheter course that may mimic malposition, arterial cannulation, or extravascular placement. The differential diagnosis for a left‐sided mediastinal catheter trajectory should also include catheterization of the left superior intercostal vein, which can similarly mimic the radiographic appearance of a PLSVC on frontal chest radiography. Distinguishing these entities is important because catheter placement in an unintended venous branch may require repositioning or removal, whereas a catheter following a confirmed PLSVC drainage pathway may be acceptable for use. Similar diagnostic confusion has been described during pacemaker and defibrillator lead placement, further emphasizing the importance of recognizing PLSVC before manipulating or repositioning intravascular devices [5].

This case underscores the importance of correlating procedural findings with postprocedure imaging when evaluating unexpected catheter positioning following left‐sided venous access. Catheter removal based solely on radiographic appearance may expose patients to unnecessary repeat procedural risk, including vascular injury, infection, and delay in delivery of critical therapies [6]. Although the present case involves a central venous catheter rather than an implanted cardiac device, the same procedural risk‐management principle applies: unnecessary device manipulation, catheter exchange, or repeat instrumentation should be avoided when safe alternatives exist, as these interventions may increase the risk of infection, vascular injury, and other device‐related complications [7]. Early radiology consultation and review of prior imaging can help identify underlying venous anatomic variants, allowing clinicians to recognize atypical but acceptable catheter positions and make informed decisions regarding catheter use.

Beyond the anatomic implications, PLSVC raises theoretical pharmacokinetic considerations relevant to anesthesiologists. Certain intravenously administered medications undergo significant first‐pass pulmonary uptake or metabolism, including opioids such as fentanyl [8] and local anesthetics such as lidocaine [9]. In this case, because the PLSVC drained into the coronary sinus and subsequently the right atrium, medications administered through the catheter would still be expected to pass through the pulmonary circulation before reaching the systemic circulation, making clinically significant alteration in first‐pass pulmonary drug extraction unlikely. In contrast, PLSVC drainage directly into the left atrium could create a right‐to‐left venous pathway, allowing medications, air, or particulate matter administered through left‐sided venous access to bypass the pulmonary circulation and enter the systemic circulation directly. At present, there are no clinical data supporting routine dose adjustment in patients with PLSVC; however, awareness of this anatomic variant may be relevant when interpreting unexpected drug responses in complex perioperative settings.

When an unexpected left‐sided mediastinal catheter course is identified after left internal jugular or subclavian venous access, management should be systematic. First, assess for immediate safety concerns by confirming catheter function, venous‐appearing nonpulsatile blood return, and absence of clinical evidence of arterial, pleural, or extravascular placement. Second, correlate the chest radiograph with prior or current cross‐sectional imaging when available, specifically looking for a duplicated superior vena cava, absence of expected midline crossover, drainage toward the coronary sinus, and alternative explanations such as cannulation of the left superior intercostal vein. Third, determine the drainage pathway. A catheter within a PLSVC draining to the coronary sinus and right atrium may be acceptable for use if it functions appropriately and no complications are present, whereas drainage into the left atrium or placement within an unintended small venous branch should prompt avoidance, repositioning, or removal. Early radiology consultation can help prevent unnecessary catheter manipulation or repeat instrumentation.

Recognition of PLSVC during left‐sided central venous access is essential to prevent unnecessary catheter manipulation, repeat instrumentation, and escalation of diagnostic evaluation. Familiarity with this congenital venous variant allows anesthesiologists and intensivists to interpret atypical imaging accurately and manage vascular access safely.

## Funding

No external funding was received.

## Ethics Statement

This activity was reviewed by the University of Tennessee Medical Center Institutional Review Board and determined to meet criteria for a case report not constituting human subjects research (IRB Case Report CR#658).

## Consent

Written informed consent and HIPAA authorization for publication of this case report and accompanying images were obtained from the patient’s legally authorized representative.

## Conflicts of Interest

The authors declare no conflicts of interest.

## Supporting Information

Additional supporting information can be found online in the Supporting Information section.

## Supporting information


**Supporting Information** CARE‐checklist‐English‐2013.

## Data Availability

The data that support the findings of this study are available on request from the corresponding author. The data are not publicly available due to privacy or ethical restrictions.
